# The Synthesis and Anti-Cytomegalovirus Activity of Piperidine-4-Carboxamides

**DOI:** 10.3390/v14020234

**Published:** 2022-01-25

**Authors:** Xin Guo, Ayan Kumar Ghosh, Robert F. Keyes, Francis Peterson, Michael Forman, David J. Meyers, Ravit Arav-Boger

**Affiliations:** 1 Department of Pediatrics, Division of Infectious Disease, Medical College of Wisconsin, Milwaukee, WI 53226, USA; xinyuanlian@163.com (X.G.); aghosh@mcw.edu (A.K.G.); 2 Department of Biochemistry, Medical College of Wisconsin, Milwaukee, WI 53226, USA; rkeyes@mcw.edu (R.F.K.); fpeterso@mcw.edu (F.P.); 3 Department of Pathology, Johns Hopkins University School of Medicine, Baltimore, MD 21287, USA; mformaa@jhmi.edu; 4 Department of Pharmacology and Molecular Sciences, Johns Hopkins University School of Medicine, Baltimore, MD 21205, USA

**Keywords:** cytomegalovirus, mouse cytomegalovirus, piperidine-4-carboxamides, add-on removal

## Abstract

Treatment options for human cytomegalovirus (CMV) remain limited and are associated with significant adverse effects and the selection of resistant CMV strains in transplant recipients and congenitally infected infants. Although most approved drugs target and inhibit the CMV DNA polymerase, additional agents with distinct mechanisms of action are needed for the treatment and prevention of CMV. In a large high throughput screen using our CMV-luciferase reporter Towne, we identified several unique inhibitors of CMV replication. Here, we synthesize and test in vitro 13 analogs of the original NCGC2955 hit (**1**). Analogs with no activity against the CMV-luciferase at 10 µM and 30 µM (**2**–**6**, **10**–**14**) were removed from further analysis. Three analogs (**7**–**9**) inhibited CMV replication in infected human foreskin fibroblasts. The EC_50_ of (**1**) was 1.7 ± 0.6 µM and 1.99 ± 0.15 µM, based on luciferase and plaque assay, respectively. Compounds **7**, **8**, and **9** showed similar activities: the EC_50_ values of **7** were 0.21 ± 0.06 µM (luciferase) and 0.55 ± 0.06 (plaque), of **8**: 0.28 ± 0.06 µM and 0.42 ± 0.07, and of **9**: 0.30 ± 0.05 µM (luciferase) and 0.35 ± 0.07 (plaque). The CC_50_ for **7**, **8**, and **9** in non-infected human foreskin fibroblasts was > 500µM, yielding a selectivity index of >1500. Compounds **1**, **7**, and **8** were also tested in CMV-infected primary human hepatocytes and showed a dose–response against CMV by luciferase activity and viral protein expression. None of the active compounds inhibited herpes simplex virus 1 or 2. Compounds **7** and **8** inhibited mouse CMV replication in vitro. Both inhibited CMV at late stages of replication; **7** reduced virus yield at all late time points, although not to the same degree as letermovir. Finally, the activity of analog **8** was additive with newly identified CMV inhibitors (MLS8969, NFU1827, MSL8554, and MSL8091) and with ganciclovir. Further structural activity development should provide promising anti-CMV agents for use in clinical studies.

## 1. Introduction

Infection with human cytomegalovirus (CMV), a member of the herpesvirus family, is common in humans. The seroprevalence rates increase with age, reaching 90% in individuals older than 80 years [1]. CMV establishes lifelong persistent infection, and patients typically remain asymptomatic. In immunocompromised hosts, including transplant recipients and patients with AIDS, CMV causes significant morbidity and mortality [2,3,4,5].

CMV is the most common congenital infection worldwide [6,7,8]. It is the leading infectious cause of hearing loss and central nervous system damage in children.

Most of the systemic anti-CMV drugs target the viral DNA polymerase [9,10,11,12]. Their use is associated with considerable toxicities to the bone marrow (ganciclovir (GCV)) and kidneys (foscarnet and cidofovir), and the emergence of resistant viruses [9,10,11,12]. A phase III clinical trial documented the prevention of hearing loss in congenitally infected children treated with intravenous GCV, paving the way for the treatment of congenital CMV with central nervous system involvement [13]. A follow-up phase III clinical trial of oral valganciclovir (the valyl-ester prodrug of GCV) suggested modest neurobehavioral benefit for six months, compared to six weeks of therapy [14]. A six-weeks course of valganciclovir may not prevent the development of long-term hearing loss, but it needs to be determined whether longer courses may have an effect on hearing preservation [15], and how common GCV-resistant mutants are identified in these children. 

The widespread use of a limited number of drugs often leads to the development of drug-resistant CMV strains [16,17]. Letermovir, a terminase inhibitor, is approved for CMV prophylaxis after hematopoietic stem cell transplantation [18,19], and resistance has already been reported [20]. Maribavir targets the viral UL97 kinase and showed promising results [21,22]. A phase II clinical trial of maribavir showed ~65% response across all doses. There were no major safety issues and no bone marrow suppression, but CMV recurrences occurred in 35% of the participants [23]. In a phase III clinical trial, maribavir was superior to conventional antiviral therapy for resistant/refractory CMV. This led to the FDA approval of maribavir for adults and children (12 years of age and older) with CMV infection/disease that is refractory to treatment with ganciclovir, valganciclovir, cidofovir or foscarnet. The overall problems of toxicity, resistance, oral-bioavailability, and high-cost drive CMV drug discovery. We recently reported on a successful completion of the largest high-throughput screen (HTS) of ~400,000 compounds that resulted in the identification of five structurally unique CMV inhibitors, active at low µM concentrations [24]. One of these compounds was NCGC2955, which was selected for further development against CMV. Structurally related compounds to NCGC2955, which all contained the piperidine-4-carboxamide motif (see purple rectangle for Compound **1**, Table 1), were reported to inhibit neurotropic alphaviruses (RNA viruses) [25,26]. Here, we report structure-activity relationship (SAR) studies to further characterize the anti-CMV activities of the parental NCGC2955 (**1**) and several structurally related analogs. 

## 2. Materials and Methods

### 2.1. Compounds

Compound synthesis and characterization is detailed in the Supplementary Material. The purities of all the synthesized compounds used in the bioassays were determined by HPLC using either a Phenomenex Luna C18 3.0 × 75 mm column with a 7 min gradient of 4–100% ACN in H_2_O with 0.05% *v*/*v* TFA, or a Higgins Analytical, Inc. Targa C18 5 µm 4.6 × 150 mm column with a 30 min gradient of 0–100% ACN in H_2_O with 0.01% TFA, and either the absorbance detection at 254 nm or evaporative light scattering detection. All the compounds used in the bioassays exhibited NMR and MS data consistent with their structures and purities of >98% as determined by RP-HPLC.

### 2.2. Viruses

A pp28-luciferase recombinant Towne CMV strain was used in the luciferase assays. The virus expressed luciferase under the control of the UL99 (pp28) late promoter, and was reported to provide a sensitive and reproducible reporter for drug screening [27]. CMV Towne (VR-977) was used for the Western blots. The CMV UL32-EGFP-TB40 [28] (VR-1578) was obtained from ATCC (VR-1578). Clinical isolates of Human Herpesvirus 1 and 2 (HSV1, HSV2) were collected from the Johns Hopkins Microbiology laboratory without identifiers that could link them to a specific patient. Mouse embryonic fibroblasts (MEFs; ATCC, CRL-1658) were used for infection with the MCMV Smith strain (ATCC VR-1399).

### 2.3. Cell Culture, Virus Infection, and Anti-Viral Assays

Human foreskin fibroblasts (HFFs), passage 12 to 16 (ATCC, CRL-2088), were grown in Dulbecco’s modified Eagle medium (DMEM), containing 10% fetal bovine serum (FBS) (Gibco, Carlsbad, CA, USA) in a 5% CO_2_ incubator at 37 °C. Vero cells (Vervet monkey kidney epithelial cells) were from ATCC, CCL-81. Virus infection and anti-viral assays followed previously reported protocols for the lab [24]. Infection was carried out at a multiplicity of 1 plaque forming unit (PFU)/cell (MOI = 1 PFU/cell), unless otherwise noted. Following 90 min adsorption, media containing the virus was removed and replaced by DMEM with 4% FBS (Gibco) in the presence or absence of compounds. Infected or infected-treated HFFs were collected at specific time points depending on the assay used. In the luciferase assay, cell lysates were collected at 72 hpi and the luciferase activity was measured using the Glomax-Multi + Detection System (Promega, Madison, WI, USA), as previously described [27]. For plaque assays, HFFs were seeded into 12-well plates (2 × 10^5^ cells/well) and infected with CMV TB40 at approximately 150 plaques/well. After 90 min, media were aspirated, and DMEM containing 0.5% carboxymethyl-cellulose (CMC), 4% fetal bovine serum (FBS), and compounds were added into duplicate wells. Following incubation at 37 °C for 7–10 days, the overlay was removed and plaques were counted after crystal violet staining. For HSV1 and HSV2 replication in Vero cells (100 plaques/well), the adsorption time was 60 min and plaques were counted after 48 h. For MCMV replication in MEFs (100 plaques/well), the adsorption time was 90 min and plaques were counted after 72 h.

Primary human hepatocytes (PHHs, PXB-cells) isolated by the collagenase perfusion method from chimeric urokinase-type plasminogen activator/severe combined immunodeficiency (uPA/SCID) mice with humanized livers that were obtained from PhoenixBio (Hiroshima, Japan). The cells were seeded on type I collagen-coated 96-well plates or in 24-well plates at a density of 6.8 × 10^4^ and 4 × 10^5^ cells/well. The cells were washed with dHCGM media substituted with 10% FBS and allowed to recover in the complete media for 24 h. For the maintenance of the PHHs, the dHCGM media (10% FBS) was changed every 3–4 days. The PHHs in the 96-well plates were infected with pp28-luciferase CMV or CMV Towne at MOI = 1 PFU/cell for 90 min in serum-free dHCGM media. After infection, the cells were washed once and incubated with several concentrations of GCV, Compounds **1**, **7**, and **8** diluted in dHCGM media containing 10% FBS for 96 h. The cells were lysed in a cell culture lysis buffer (Promega) containing protease inhibitors and the luciferase activity was measured. Lysates from the 96-well plate were also used for the analysis of viral protein expression by Western blot.

### 2.4. Toxicity Assays

A 3-(4,5-dimethyl-2-thiazolyl)-2,5-diphenyl-2H-tetrazolium bromide (MTT) assay was performed according to the manufacturer’s instructions (Millipore Sigma). Non-infected cells were treated with NCGC2955, **7**, **8**, **9** for 72 h, and 20 μL/well of MTT ([3-(4,5-Dimethyl-2-thiazolyl)-2,5-diphenyl-2H-tetrazolium bromide]), 5 mg/mL in phosphate-buffered saline (PBS) was added to each well. After shaking at 150 rpm for 5 min, the plates were incubated at 37 °C for 2–3 h. The conversion of the yellow solution to dark blue formazan by mitochondrial dehydrogenases of living cells was quantified by measuring the absorbance at 560 nm.

### 2.5. SDS-PAGE and Immunoblot Analysis

The cell lysates containing an equal amount of protein were mixed with an equal volume of sample buffer (125 mM Tris-HCl, pH 6.8, 4% SDS, 20% glycerol, and 5% *β*-mercaptoethanol) and heated for 10 min at 100 °C. Denatured proteins were resolved by tris-glycine polyacrylamide gels (8–10%) and transferred to polyvinylidine difluoride (PVDF) membranes (Bio-Rad Laboratories, Hercules, CA, USA) by electroblotting. Membranes were incubated in blocking buffer (5% *w*/*v* non-fat dry milk and 0.1% Tween-20 in PBS (PBST)) for 1 h, washed with PBST, and incubated with primary antibodies at 4 °C overnight. Membranes were washed with PBST and incubated with horseradish peroxidase-conjugated secondary antibodies in PBST for 1 h at room temperature. Following washing with PBST, protein bands were visualized by chemiluminescence using SuperSignal West Dura and Pico reagents (Pierce Chemical, Rockford, IL, USA). The following antibodies were used: mouse monoclonal anti-CMV IE1 and IE2 (MAB810, Millipore, Billerica, MA, USA); mouse monoclonal anti-CMV UL83 (pp65, Vector Laboratories Inc., Burlingame, CA, USA); mouse monoclonal anti-CMV UL84; and mouse anti-β-actin anti-mouse IgG (Santa Cruz Biotechnology, Santa Cruz, CA, USA). Horseradish peroxidase (HRP)-conjugated anti-mouse IgG was from GE Healthcare (Waukesha, WI, USA).

### 2.6. DNA Isolation and Quantitative Real-Time (qPCR)

Total DNA was isolated from non-infected and CMV-infected HFFs using the Wizard SV genomic DNA isolation kit (Promega, Madison, WI, USA). To determine the viral load in the supernatant, total DNA was isolated from supernatants using automated DNA extraction on a BioRobot M48 instrument (Qiagen, Valencia, CA, USA). A US17 real-time PCR assay that targets 151-bp from the highly conserved US17 region of the CMV genome was used [29]. The primers and probe used for US17 were: forward 5′-GCGTGCTTTTTAGCCTCTGCA-3′, reverse 5′-AAAAGTTTGTGCCCCAACGGTA-3′, and US17 probe FAM-5′ TGATCGGGCGTTATCGCGTTCT-3′.

### 2.7. Add-On and Removal Assays

In the add-on group, compounds were added to infected HFFs at 0, 6, 24, and 48 hpi, and the luciferase activity was measured at 72 hpi. In the removal group, compounds were added immediately after virus infection and were subsequently removed after 0, 6, 24, and 48 h; luciferase was measured at 72 hpi.

### 2.8. Combination Assays

These experiments were performed as previously reported [30]. The combination of Compound **8** and each compound was tested using the pp28-luciferase CMV. Briefly, 2 × 10^6^ HFFs/plate were seeded in 96-well plates and infected with the pp28-luciferase CMV strain (MOI = 1 PFU/cell). First, a dose–response curve was generated for each compound individually to determine its EC_50_ value. Then, the compounds were combined at twice their EC_50_ values, diluted in DMEM with 4% FBS, followed by serial dilution, and added in combination after infection. The luciferase activity of each compound individually and in combination was quantified at 72 hpi. The Bliss model was used to calculate the effect of each drug combination on pp28-luciferase activity (Kapoor/Ghosh JMC). In this model, synergistic compounds will yield a ratio > 1 of the observed fold inhibition divided by the expected fold inhibition. Antagonistic compounds will yield a ratio < 1. Additive compounds will yield a ratio = 1.

### 2.9. Statistical Analysis

Dose-response curves were generated as previously described [30]. The EC_50_ and CC_50_ values were calculated using GraphPad Prism software using the non-linear curve fitting and the exponential form of the median effect equation, where the percent inhibition = 1/[1 + (CC_50_ or EC_50_/drug concentration)m], where m is a parameter that reflects the slope of the concentration-response curve. The statistical significance between two groups was analyzed by the two-tailed Student’s *t*-test, and the asterisks indicate the statistical significance: *, *p* < 0.05; **, *p* < 0.01; and *** *p* < 0.001. All experiments were performed at least twice.

## 3. Results 

### 3.1. CMV Inhibition by NCGC2955 Analogs

We first investigated the importance of the isopropyl carboxamide found in NCGC2955 1, (Table 1). Moving the isopropyl carboxamide from the 4-position of the piperidine found in Compound **1** to the 3-position of the piperidine in Compound 2 (isopropyl carboxamide in red) resulted in the loss of anti-CMV activity (Table 1). Since the synthesis of the thieno[3,2-b]pyrrole heterocycle found in **1** required the synthesis of potentially explosive azido precursors on gram scale, we opted to test the more readily available truncated pyrrole **4** or indole **3** replacements. Both modifications (Compounds **3** and **4**) resulted in a loss of anti-CMV activity even at 30 µM (Table 1).

**Figure 1:**
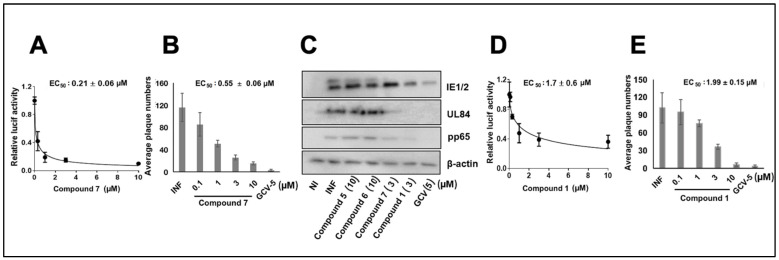
**CMV inhibition with Compounds 1 and 7 in HFFs**. (**A**) HFFs were infected with pp28-luciferase CMV (MOI = 1 PFU/cell) and treated with the indicated concentrations of Compound **7**. Luciferase activity was measured in cell lysates at 72 hpi. The data represent mean values (±SD) of triplicate determinations from two independent experiments. (**B**) HFFs were infected with CMV TB40 (150 PFU/well) and treated with the indicated concentrations of **7**. Plaques were stained and counted at day 7 post infection. The data represent mean values (±SD) of the triplicate determinations from two independent experiments. (**C**) HFFs were infected with CMV Towne and treated with Compounds **1, 5**, **6**, **7**, and GCV at the indicated concentrations in parentheses. An immunoblot was performed for the detection of viral proteins at 72 hpi. The experiment was repeated three times, and data from a single representative experiment are shown. (**D**) HFFs were infected with pp28-luciferase CMV and treated with the indicated concentrations of Compound **1**. Luciferase activity was measured in cell lysates at 72 hpi. The data represent mean values (±SD) of triplicate determinations from two independent experiments. (**E**) HFFs were infected with CMV TB40 (150 PFU/well) and treated with the indicated concentrations of Compound **1**. Plaques were stained and counted at day 7 post infection.

The compounds with structural similarity to **1** were reported as inhibitors of neurotropic alphaviruses (RNA viruses) [25,31,32], therefore the pyrrole 4-(2-aminoethyl)pyridine **7** [26] was resynthesized (4-(2-aminoethyl)pyridine colored blue). Three analogs of Compound **1** (**5–7**) were initially tested. Compounds **5** and **6** did not inhibit the pp28-luciferase CMV at concentrations ranging from 3–30 µM (Table 1). However, compound **7** exhibited the inhibition of pp28-luciferase CMV in a dose-dependent manner, with EC_50_ of 0.21 ± 0.06 µM (Figure 1A). In a plaque assay using the CMV TB40 strain, **7** also displayed dose-dependent activity, with EC_50_ of 0.55 ± 0.06 µM (Figure 1B). A Western blot analysis performed on cell lysates collected at 72 h post infection (hpi) with CMV Towne, showed no reduction in the level of pp65, UL84, or IE1/2 after treatment with analogs **5** and **6** (10 µM), while treatment with **7** and NCGC2955 resulted in a significant inhibition of protein expression. GCV treatment (5 µM), as expected, reduced the level of CMV proteins (Figure 1C). The activity of NCGC2955 **1** was measured in the same set of experiments and revealed an EC_50_ of 1.7 ± 0.6 µM, and 1.99 ± 0.15 µM for luciferase inhibition and plaque reduction, respectively (Figure 1D,E).

**Figure 2:**
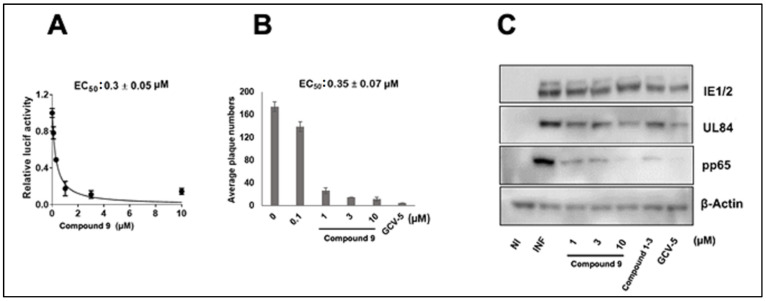
Anti-CMV activity of Compound **9** in HFFs. (**A**) HFFs were infected with pp28-luciferase CMV (MOI = 1 PFU/cell) and Compound **9** was added at the indicated concentrations after infection. Luciferase activity was measured in cell lysates at 72 hpi. The data represent mean values (±SD) of triplicate determinations from two independent experiments. (**B**) HFFs were infected with CMV TB40 (150 PFU/well) and treated with the indicated concentrations of Compound **9**. Plaques were stained and counted at day 7 post infection. The data represent mean values (±SD) of the triplicate determinations from two experiments. (**C**) HFFs were infected with CMV Towne and treated with Compound **9** or GCV at the indicated concentration. The level of viral proteins was measure by Western blot at 72 hpi. The experiment was repeated three times, and data from a single representative experiment are shown. NI—noninfected, I—Infected, and GCV—ganciclovir.

The overall anti-CMV activity of **7**, which contains a 4-(2-aminoethyl) pyridine amide, was improved compared to **1**, which contains an isopropyl amide. Compound **7** differed from **5** and **6** only in the position of nitrogen in the pyridine ring, suggesting that the nitrogen in Compound **7** forms a specific interaction with its target. Truncated analogs of **7** (Compounds **10**–**12**), which differ by the removal of one methylene spacer also lost anti-CMV activity, suggesting that an optimal distance between the 4-carboxy piperidine and the 4-pyridine is required for CMV inhibition (Table 1). A close analog of **7**, Compound **9**, also showed anti-CMV activity in both luciferase and plaque assay (Figure 2, Table 1), EC_50_ 0.3 ± 0.05 µM and 0.35 ± 0.07 µM, respectively, suggesting that the replacement of the pyrrole with thienopyrrole has little effect on CMV inhibition. The CC_50_ of Compounds **7** and **9** was >500 µM, yielding a selectivity index of >1500.

The data represent mean values (±SD) of the triplicate determinations from two independent experiments. NI—noninfected, I—Infected, and GCV—ganciclovir.

### 3.2. CMV Inhibition by Pyridine Analogs of NCGC2955

**Figure 3:**
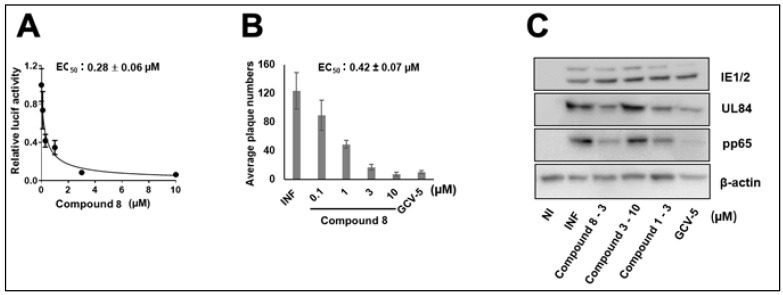
CMV inhibition with Compound **8** in HFFs. (**A**) HFFs were infected with pp28-luciferase CMV, and the indicated concentrations of Compound **8** were added after infection. Luciferase activity was measured in cell lysates at 72 hpi. The data represent mean values (±SD) of the triplicate determinations from two independent experiments. (**B**) HFFs were infected with CMV TB40 (150 PFU/well) and treated with the indicated concentrations of Compound **8**. Plaques were stained and counted at day 7 post infection. The data represent mean values (±SD) of triplicate determinations from two independent experiments. (**C**) HFFs were infected with CMV Towne and treated with Compounds **1**, **8**, **3**, and GCV at the indicated concentrations. The expression of viral proteins was determined by Western blot at 72 hpi. The experiment was repeated three times, and data from a single representative experiment are shown. NI—noninfected, I—Infected, and GCV—ganciclovir.

Several pyridine analogs were next tested against CMV. Compound **8** showed dose-dependent activity against CMV, EC_50_^−^ 0.28 ± 0.06 (luciferase, Figure 3A), and 0.42 ± 0.07 (plaque, Figure 3B), while Compound **3** had no activity against CMV in pp28- luciferase assay. Similarly, the viral proteins UL84 and pp65 were reduced following treatment with Compound **8** and the original Compound **1** (3 µM), while Compound **3** showed no inhibitory activity at 10 µM (Figure 3C). GCV (5 µM) inhibited IE2, UL84, and pp65 to a higher degree than Compound **1** and analog **8**, suggesting the activity of these compounds may occur at a later stage of CMV replication.

### 3.3. NCGC2955 Analogs Inhibit CMV in Primary Human Hepatocytes

**Figure 4:**
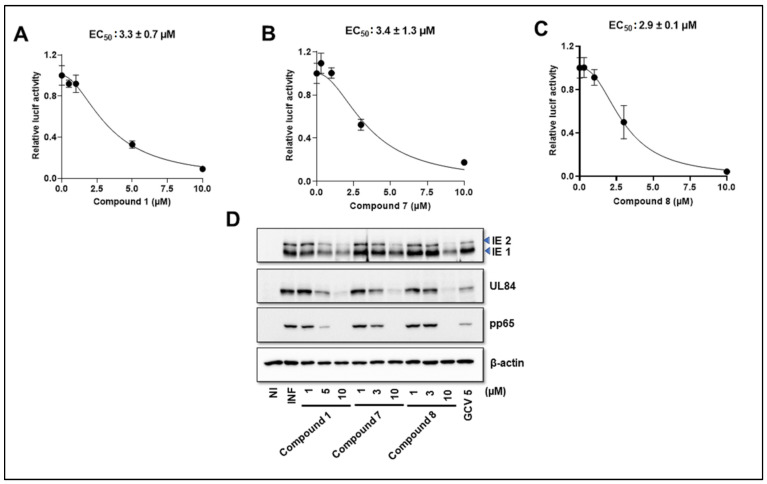
Activity of Compounds **1**, **7**, **8** in CMV-infected primary human hepatocytes. (**A**–**C**) Primary human hepatocytes (PXB-cells) were infected with pp28-luciferase CMV (MOI =1 PFU/cell) and treated with the indicated concentrations of Compounds **1**, **7**, and **8**. Luciferase activity was measured in cell lysates at 72 hpi. The data represent mean values (±SD) of the triplicate determinations from two independent experiments. (**D**) Primary human hepatocytes were infected with CMV Towne and treated with Compounds **1, 7**, **8**, and GCV at the indicated concentrations. An immunoblot was performed for the detection of viral proteins at 72 hpi. Data from a single experiment are shown. NI—noninfected, I—Infected, and GCV—ganciclovir.

To expand our studies to another clinically relevant cell line, the activity of Compounds **1**, **7**, **8** was tested in CMV-infected primary human hepatocytes. The pp28-luciferase CMV was used for infection, and luciferase activity was measured at 72 hpi. All three compounds showed dose-dependent activity with a similar EC_50_ of ~3 µM (Figure 4A–C). Viral proteins were also reduced with the three compounds (Figure 4D).

### 3.4. NCGC2955 Analogs Inhibit Mouse CMV (MCMV) but Not Herpes Simplex Virus 1 or 2 (HSV1 or 2)

**Figure 5:**
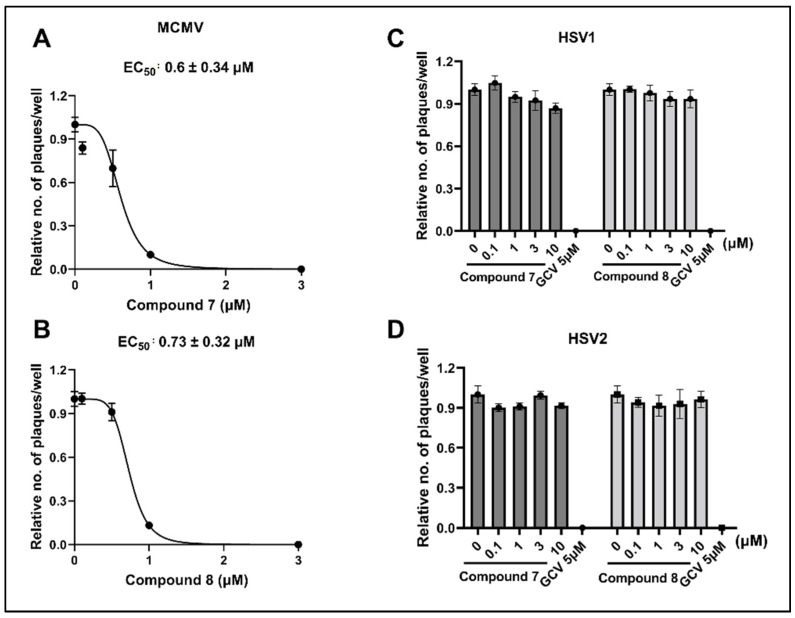
Activity of Compounds **7** and **8** against MCMV, HSV1 and HSV2. (**A**,**B**) Mouse embryonic fibroblasts were infected with MCMV at 100 PFU/well and treated with the indicated concentrations of **7** and **8**. Infected cells were stained, and the viral plaques were counted at 72 hpi. The dose-response curves represent the relative fold inhibition and the data points represent mean ± SD from two independent experiments. (**C**,**D**) Vero cells were infected with HSV1 and HSV2 (100 PFU/well) and treated with the indicated concentrations of Compounds **7** and **8**. Infected cells were stained and the viral plaques were counted at 36 h and 24 h post infection for HSV1 and HSV2, respectively. The data set represent mean ± SD from two independent experiments.

To evaluate the antiviral activity of these compounds for future in vivo studies, and to determine whether other herpesviruses were inhibited, Compounds **7** and **8** were tested against MCMV and HSV1/2, respectively. Both analogs inhibited MCMV in vitro at sub-micromolar concentrations, and the EC50 values were 0.6 ± 0.34 µM and 0.73 ± 0.32 µM, respectively (Figure 5A,B). None of the analogs tested showed any activity against HSV1 or HSV2, similar to the NCGC2955 (Figure 5C,D) [24]. GCV (5 µM), used as a positive control, showed complete inhibition of HSV1 and HSV2.

### 3.5. NCGC2955 Analogs Are Late Inhibitors of CMV Replication

**Figure 6:**
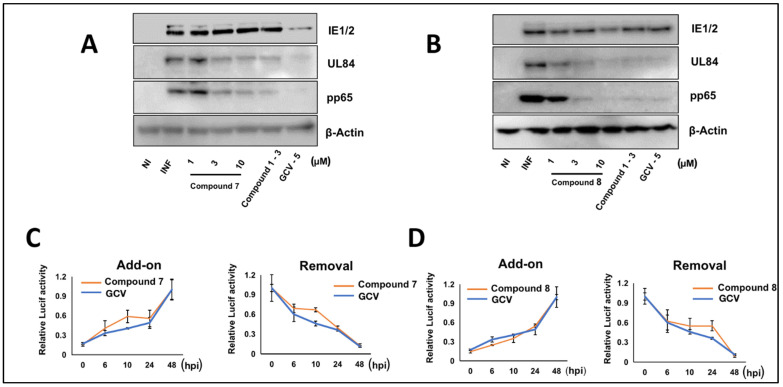
Effect of Compounds **7** and **8** on CMV protein expression and timing of activity. (**A**,**B**) HFFs were infected with CMV Towne (1 PFU/cell) and treated with Compounds **1**, **7**, **8**, and GCV at the indicated concentrations. The expression of viral proteins was determined by Western blot at 72 hpi. The experiment was repeated three times, and data from a single representative experiment are shown. (**C**,**D**) HFFs were infected with pp28-luciferase CMV (MOI = 0.1 PFU/cell). Compounds **7**, **8** (3 µM), or GCV (5 µM) were either added or removed at different times after infection (0, 6, 24, 48, 72 h). Luciferase activity was measured in cell lysates at 72 hpi. Data shown are the average of three independent experiments (average ± SD).

The timing of activity of analogs **7** and **8** was evaluated by Western blot (Figure 6A,B) and compound addition or removal at different times during infection (Figure 6C,D). The effect of GCV on reducing the level of viral proteins was similar to but more significant than **7** (Figure 6A,B). Analogs **7** and **8** were tested when added or removed at 6, 10, 24, and 48 hpi. Although the timing of maximal CMV inhibition of **7** and **8** overlapped with that of GCV, since a difference was observed in the viral protein expression, experiments at later time points of the infection were performed.

### 3.6. NCGC2955 Analogs Reduce CMV Yield

**Figure 7:**
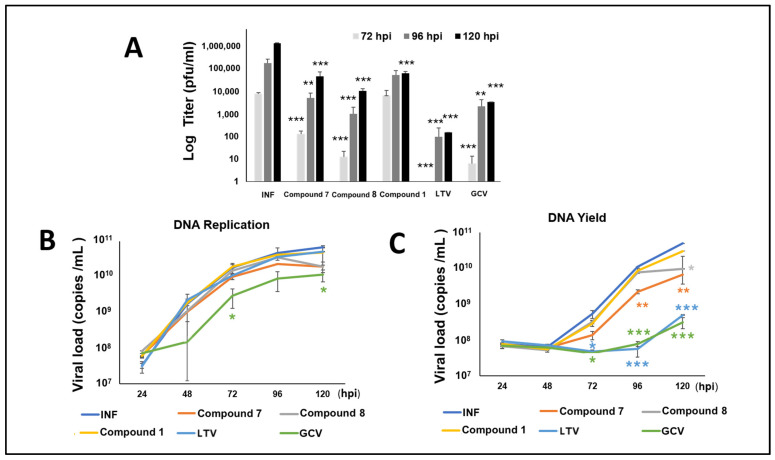
Effect of Compounds **1**, **7**, and **8** on viral progeny, DNA replication (infected cells), and DNA yield (supernatants). HFFs were infected with CMV TB40 (150 PFU/well) and treated with Compounds **1**, **7, 8** (all at 3 µM), LTV (10 nM), and GCV (5 µM). DNA was isolated from the infected cells, and supernatants were harvested at the indicated time points. (**A**) Titration of supernatants was performed by plaque assay. The plaques were stained and counted at day 7 post infection. The data represent the mean values (±SD) of the triplicate determinations from two independent experiments. (**B**,**C**) Viral DNA replication in cells (**B**) and viral DNA load in the supernatants (**C**) were determined by real-time PCR. Data shown are average ± SD of quadruplicate values from two independent experiments. *, *p* < 0.05; **, *p* < 0.01; and *** *p* < 0.001.

To better define the timing of CMV inhibition by the new analogs, HFFs were infected with CMV TB40 and treated with Compounds **1**, **7**, or **8** (all at 3 µM), letermovir (10 nM), and GCV (5 µM). The supernatants from the infected cells were harvested at 72, 96, and 120 h, and titered by plaque assay. The plaques were stained and counted at day 7 post infection. The reduction in viral titer with **7** and **8** was higher than with **1** and overall similar to GCV. Letermovir (LTV) showed the strongest reduction in viral progeny at all time points (Figure 7A). Viral DNA replication was measured in cells and supernatants (Figure 7B,C) by real-time PCR of CMV US17. GCV reduced DNA replication in both cells and supernatants, while LTV reduced viral loads in supernatants. Compounds **1**, **7**, and **8** did not reduce the cellular viral loads, but **7** decreased the viral loads in supernatants at all time points and **8** decreased viral loads at 120 hpi.

### 3.7. NCGC2955 Analogs Are Additive with Newly Identified CMV Inhibitors and GCV

**Figure 8:**
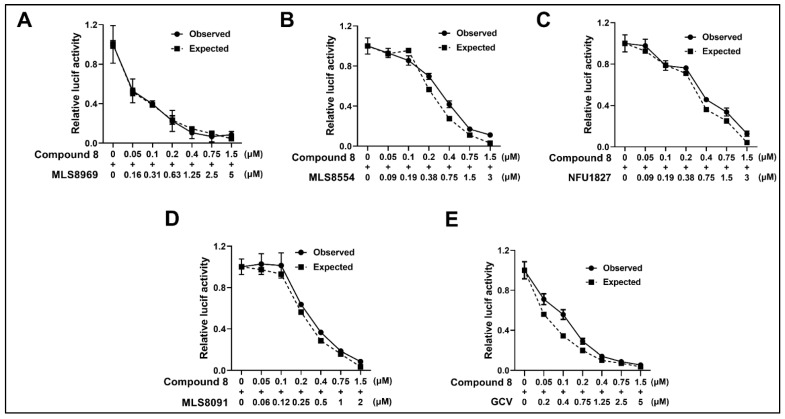
Combination of Compound **8** is an additive with newly identified CMV inhibitors and GCV. (**A**–**E**) HFFs were infected with pp28-luciferase CMV (MOI = 1 PFU/cell) and first treated with each individual compound, followed by a combination of each compound with **8** at different concentrations. In the drug combination experiments, CMV-infected HFFs were treated with an initial drug concentration of twice the EC_50_ value of the individual compound and two-fold serial dilutions. Luciferase activity was measured in cell lysates at 72 hpi and the Bliss model was used to calculate the anti-CMV activity of the compounds in their combination. Solid lines indicate the observed CMV inhibition (dose–response) and dotted lines indicate the expected CMV inhibition at each dose of drug combination. The experiments were repeated twice. Data from a single representative experiment are shown.

Analog **8** was tested in combination with newly identified CMV inhibitors MLS8969, NFU1827, MLS8554, and MLS8091, and GCV [24]. All combination experiments revealed that **8** was additive with all tested compounds with a calculated Bliss coefficient ranging from 1–1.3 (Figure 8, Table 2), suggesting an independent mode of action.

## 4. Discussion

Hit NCGC2955 (Compound **1**) was identified from a recent high-throughput screen (>400,000 compounds) for human CMV inhibitors [24]. It showed in vitro activity against TB40, ganciclovir resistant pp28-luciferase CMV, and mouse CMV at low µM concentrations, but had no activity against HSV1 or 2. The structurally related compounds of Compound **1** were reported to inhibit RNA viruses [25,26]. Piperidine-4-carboxamide analogs were studied in detail in cell-based assays using the Western Equine Encephalitis Virus (WEEV), replicons and showed half-maximal inhibitory concentrations of ~1 µM and selectivity indices of >100. CCG205432, similar to our Compound **8** (the TFA salt of CCG205432), inhibited the infectious virus in cultured human neuronal cells. These compounds showed broad inhibitory activity against RNA viruses in culture, including members of the *Togaviridae*, *Bunyaviridae*, *Picornaviridae*, and *Paramyxoviridae* families. Their mechanism of action was suggested to involve a host factor that modulates cap-dependent translation. CCG205432 did not directly target WEEV RNA-dependent RNA polymerase or other viral enzyme activities that would promote the development of drug-resistant viral mutants. Despite the broad-spectrum antiviral activity, our tested compounds did not inhibit HSV1 or HSV2, suggesting a specific mechanism of action that may involve the host factors required for the efficient replication of several groups of viruses. The interest in host factors that restrict virus replication, such as the electron transport system, cytochrome P450 51, mitochondrial regulatory proteins, and autophagy, led to the identification of agents that could be further developed or repurposed for CMV therapeutics as monotherapy or combination therapy with direct-acting FDA approved antiviral agents [33,34,35,36,37].

Initially, we studied the piperidine-4-carboxamide scaffold. With the synthesis of a handful of compounds, we were able to show that pyrrole, indole, and thienopyrrole are all tolerated when coupled to the 4-(2-aminoethyl)pyridine. The H-bond acceptor in the 4-position appears to be necessary, since CMV inhibition was not observed with the placement of this moiety at the 2- or 3-position. We were also able to demonstrate that CMV inhibition was specific to the position of the pyridine nitrogen atom and spacer between the amide and pyridine.

In addition to testing our compounds in human foreskin fibroblasts, we extended our studies to include primary human hepatocytes as another clinically relevant cell line for infection with CMV. Hepatocytes have rarely been used for CMV infection [38]. These cells were infected with the pp28-luciferase CMV. The three analogs tested revealed similar activity against CMV with EC_50_ values at around 3 µM, although in HFFs their activity was improved. We noted that the variability in antiviral activity was observed in different cell systems. Maribavir showed varied EC_50_ values for CMV inhibition depending on the fibroblasts used, which was attributed to the activity of kinases in different cells [39].

Despite the inhibition of viral progeny by plaque assays and the observed decrease in viral protein levels, the influence of NCGC2955 analogs on viral DNA replication and viral DNA yield was modest (Figure 7), suggesting inhibition via novel mechanisms that do not involve the DNA replication machinery. The terminase complex, targeted by LTV, cleaves DNA to package the viral genome into the capsid [40]. Compared to NCGC2955 analogs, LTV showed a stronger reduction in viral progeny and viral loads in supernatants. These data may suggest the production of CMV DNA containing non-infectious virus that lacks some viral proteins.

Our study is the beginning of structural activity relationship (SARs), following a large high throughput screen for CMV inhibitors. Further studies, including the resistance selection and target identification along with structure-based design, may lead to the identification of novel CMV inhibitors for in vivo studies. Our future goals include increasing the anti-CMV potency and further exploring the SARs, so that a tool compound can be synthesized to aid in the identification of a viral target. This can be approached by varying the H-bond accepting potential of the pyridine. Further SARs will be developed around the chain length. Additionally, the piperidine will be replaced with pyrrolidine and azetidine to fine tune the optimal length and angle of the pyridine’s interaction in the active site. The chain length of the 4-chlorobenzyl will also be explored as well as the electronics of the ring. The generation of metabolically stable analogs will assist exploring the in vivo efficacy in animal models.

Conclusion: Piperidine-4-carboxamide analogs inhibit cytomegalovirus with high selectivity and are additive with GCV. Their activity in primary human hepatocytes suggests they may be active in vivo and should be further developed for CMV therapeutics.

## Figures and Tables

**Table 1:** Chemical structure of compounds **1**–**14**. Provided are the EC_50_ values for pp28-luciferase CMV, TB40, and the CC_50_ measured in non-infected HFFs. All concentrations are in µM. Comp—compound number.

Comp	Structure	EC_50_ pp28-Luciferase	EC_50_ TB40	CC_50_	Comp	Structure	EC_50_ pp28-Luciferase	EC_50_ TB40	CC_50_
1	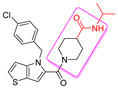	1.7 ± 0.6	1.99 ± 0.15	>200	8	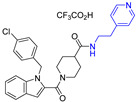	0.28 ± 0.06	0.42 ± 0.07	500
2	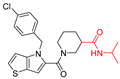	>30			9	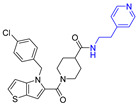	0.3 ± 0.05	0.35 ± 0.07	>500
3	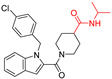	>30			10	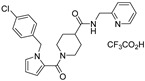	>30		
4	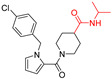	>30			11	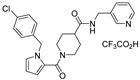	>30		
5	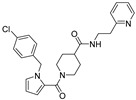	>30			12	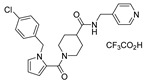	>30		
6	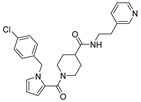	>30			13	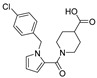	>30		
7	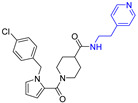	0.21 ± 0.06	0.55 ± 0.06	>500	14	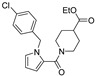	>30		

**Table 2:** EC_50_ of individual compounds and the respective Bliss coefficients.

Compound 1	EC_50_ (µM)	Compound 2	EC_50_ (µM)	Bliss Coefficient
8	0.29 ± 0.1	MLS8969	0.25 ± 0.3	1
8	0.41 ± 0.2	MLS8554	0.55 ± 0.2	1.2
8	0.35 ± 0.07	NFU1827	0.82 ± 0.1	1.2
8	0.27 ± 0.1	MLS8091	0.34 ± 0.1	1.1
8	0.38 ± 0.08	GCV	0.25 ± 0.1	1.3

## Supplementary Materials

The following supporting information can be downloaded at https://www.mdpi.com/article/10.3390/v14020234/s1, File S1: Materials and Methods.
