# Supplementary material for: The Synthesis and Anti-Cytomegalovirus Activity of Piperidine-4-Carboxamides
Source: Viruses. 2022 Jan 25;14(2):234. doi: 10.3390/v14020234 (PMC8876412; doi:10.3390/v14020234)
Supplement: Supplementary file 1 [file viruses-14-00234-s001.zip › viruses-1561488-File S1.pdf]

Materials and Methods.

General Procedures:

All commercially available reagents and solvents were used without further purification unless otherwise stated. Automated flash chromatography was performed on a Teledyne Isco CombiFlash Rf+ or Grace Reveleris using Teledyne Isco, Grace or Buchi flash silica, and/or C18 flash cartridges. Spectra were recorded on a Bruker Avance-III spectrometer (^{1}H NMR at 500 MHz and ^{13}C NMR at 125 MHz) at 296 K in $CDCl_3$ (^{1}H NMR referenced to internal standard tetramethylsilane 0 ppm, ^{13}C NMR referenced to 77.00 ppm), d6-DMSO (^{1}H NMR referenced to 2.50 ppm, ^{13}C NMR referenced to 39.510 ppm). Analytical LC-MS was performed using Agilent 1260 equipped with an autosampler (Agilent Poroshell 120 column (50 x 3.0 mm I.D., 2.7 μm); 0.05% TFA in water/acetonitrile gradient; UV detection at 215 and 254 nm) and electrospray ionization. Unless otherwise noted, all final compounds showed purity greater than 95% at 215 and 254 nm using this method. DMF=*N,N*-Dimethylformamide, HATU=1-[Bis(dimethylamino)methylene]-1H-1,2,3-triazolo[4,5-b]pyridinium 3-oxid hexafluorophosphate, R_t=retention time,

Synthesis of 4H-thieno[3,2-b]pyrrole analogs:

Scheme 1. Synthesis of Analogs 1, 2, 9.

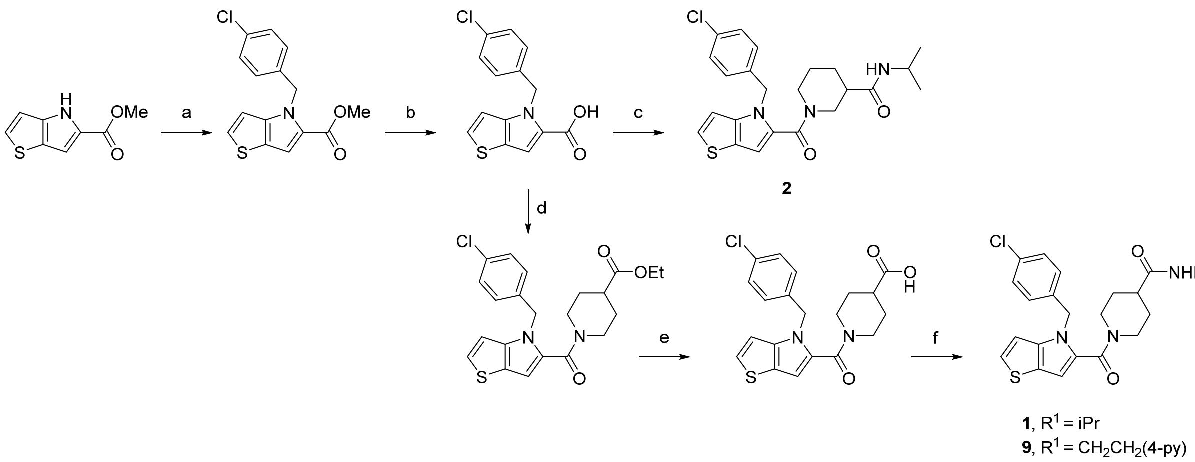

Reagents and conditions: (a) Cs_2CO_3, 4-chlorobenzyl bromide, DMF, rt (b) LiOH monohydrate, THF/MeOH/H_2O, rt (c) N-isopropylpiperidine-3-carboxamide trifluoroacetic acid salt, HATU, DIEA, DMF, rt (d) ethyl piperidine-4-carboxylate, HATU, DIEA, DMF, rt; (e) LiOH monohydrate, THF/MeOH/H_2O, rt; (f) H_2NR^1, HATU, DIEA, DMF, rt.

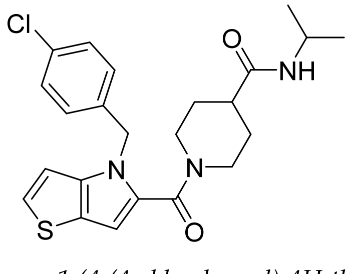

1-(4-(4-chlorobenzyl)-4H-thieno[3,2-b]pyrrole-5-carbonyl)-N-isopropylpiperidine-4-carboxamide[1] (**1**, **NCGC2955**).

[1] Kapoor, A., Ghosh, A.K., Forman, M., Hu, X., Ye, W., Southall, N., Marugan, J., Keyes, R.F., Smith, B.C., Meyers, D.J., Ferrer, M., Arav-Boger, R., 2020. Validation and Characterization of Five Distinct Novel Inhibitors of Human Cytomegalovirus. J Med Chem 63, 3896-3907.

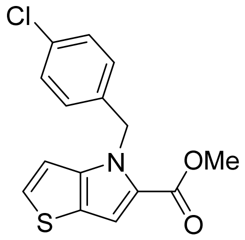

methyl 4-(4-chlorobenzyl)-4H-thieno[3,2-b]pyrrole-5-carboxylate: To a suspension of methyl 4H-thieno[3,2-b]pyrrole-5-carboxylate[2] (1.449 g, 7.99 mmol, 1.0 equiv.) and cesium carbonate (3.908 g, 11.99 mmol, 1.5 equiv.) in anhydrous DMF (10 ml) was added 4-chlorobenzyl bromide (1.97 g, 9.59 mmol, 1.2 equiv.) in one portion at rt. After stirring at rt until complete (ca. 4h), the reaction was diluted with water and extracted with EtOAc (3x20ml). The organic layers were combined, washed with brine, dried with anhydrous $MgSO_4$, and concentrated *in vacuo*. Purification by flash chromatography (3m 0%, 6m gradient 0 to 50%, 2m 50% EtOAc/Hex) provided 2.40 g of a pale yellow solid in 98% yield. ^{1}H NMR (500 MHz, CHLOROFORM-d) δ 7.33 (d, J = 5.34 Hz, 1H), 7.21 - 7.29 (m, 3H), 7.05 (d, J = 8.17 Hz, 2H), 6.85 (d, J = 5.50 Hz, 1H), 5.71 (s, 2H), 3.83 (s, 3H).

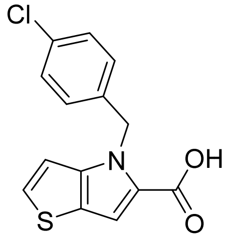

4-(4-chlorobenzyl)-4H-thieno[3,2-b]pyrrole-5-carboxylic acid: To a mixture of methyl ester **3** (3.58 g, 11.73 mmol, 1.0 equiv.) in THF (12 ml) and MeOH (12 ml) was added a solution of lithium hydroxide monohydrate in water (12 ml) in one portion. After 48 h of rapid magnetic stirring at rt, the starting material was consumed and the organic solvents were removed *in vacuo*. The remaining aqueous solution was diluted with water (ca. 20 ml) and under vigorous magnetic stirring the pH was adjusted to pH=1 with conc. aq. HCl. A precipitate formed during acidification, and was collected via vacuum filtration, washed with water and dried under vacuum to provide 3.34 g in 98% yield. ^{1}H NMR (500 MHz, DMSO-d6) δ 12.57 (br. s., 1H), 7.56 (d, J = 5.19 Hz, 1H), 7.29 - 7.43 (m, J = 8.49 Hz, 2H), 7.17 - 7.29 (m, 2H), 7.05 - 7.17 (m, J = 8.49 Hz, 2H), 5.76 (s, 2H).

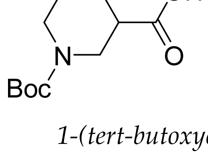

1-(tert-butoxycarbonyl)piperidine-3-carboxylic acid: To a suspension of piperidine-3-carboxylic acid (3.59 g, 27.8 mmol) and K_2CO_3 (7.68 g, 55.6 mmol, 2 equiv.) in 55 ml water was added a solution of Boc_2O (6.7 g, 30.6 mmol, 1.1 equiv.) in 14 ml of THF in small portions over 5 min. After stirring 18h at rt, the organic volatiles were removed in vacuo. The remaining aqueous solution was cooled via an ice/water bath and conc. aq. HCl was added in small portions until the pH adjusted to ca. 2-3. During this time a white solid formed and was collected via vacuum filtration to provide 3.33 g of 1-(tert-butoxycarbonyl)piperidine-3-carboxylic acid. The filtrate was extracted with EtOAc (15 ml x 3) and the organic layers were

[2] Previously synthesized according to the literature: (a) Eras, J.; Galvez, C.; Garcia, F. Reactivity of thienopyrroles. Synthesis of isomeric nitro and bromothienopyrroles. *J. Heterocycl. Chem.*, **21**, 215–217 (1984). (b) Srinivasan, S.; Schuster, G. B. A conjoined thienopyrrole oligomer formed by using DNA as a molecular guide. *Org. Lett.*, **10**, 3657–3660 (2008). (c) Ching, K.-C.; Kam, Y.-W.; Merits, A.; Ng, L.F.P.; Chai, C.L.L. Trisubstituted Thieno[3,2-b]pyrrole 5-Carboxamides as Potent Inhibitors of Alphaviruses *J. Med. Chem.*, **58**, 9196-9213, (2015).

washed with brine, dried with anhydrous $MgSO_4$, and concentrated *in vacuo* to provide an additional 2.94 g of product. ^{1}H NMR (500 MHz, DMSO-d6) δ 12.38 (br. s., 1H), 3.90 (br. s., 1H), 3.69 (br. s., 1H), 3.04 (br. s., 1H), 2.70 - 2.90 (m, 2H), 2.20 - 2.35 (m, 1H), 1.90 (d, J = 9.75 Hz, 1H), 1.61 (td, J = 3.95, 13.01 Hz, 1H), 1.43 - 1.57 (m, 1H), 1.39 (s, 9H), 1.27 - 1.37 (m, 1H).

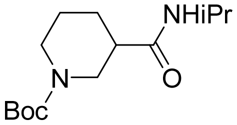

tert-butyl 3-(isopropylcarbamoyl)piperidine-1-carboxylate: To an ice/water cooled solution of 1-(tert-butoxycarbonyl)piperidine-3-carboxylic acid (1.031 g, 4.49 mmol, 1.0 equiv.) in anhydrous DMF (9 ml) was added COMU (1-Cyano-2-ethoxy-2-oxoethylidenaminooxy)dimethylamino-morpholino-carbenium hexafluorophosphate (2.12 g, 4.95 mmol, 1.1 equiv.) and N,N-diisopropylethylamine (1.76 ml, 10.12 mmol, 2.25 equiv.). After stirring for 5 min., isopropylamine (0.57 ml, 6.75 mmol, 1.5 equiv.) was added to the reaction in one portion. After stirring at rt for 21 h, the reaction was diluted with water and extracted with ethyl acetate (20 ml x 3). The combined organic layers washed with 5% aq. HCl, sat. aq. Na2CO3, brine, dried with anhydrous $MgSO_4$, and concentrated *in vacuo*. Purification by flash chromatography (0 to 100% EtOAc/Hex) followed by flash C18 purification (0 to 100% MeCN (0.05% TFA)/H2O (0.05% TFA) provided 0.97 g of a yellow solid in 79% yield. ^{1}H NMR (500 MHz, CHLOROFORM-d) δ 5.96 (br. s., 1H), 4.07 (qd, J = 6.75, 13.56 Hz, 1H), 3.78 (br. s., 1H), 3.57 (br. s., 1H), 3.32 (br. s., 1H), 3.16 (br. s., 1H), 2.24 (br. s., 1H), 1.95 (br. s., 1H), 1.83 (br. s., 1H), 1.66 (br. s., 2H), 1.47 (s, 9H), 1.08 - 1.19 (m, 6H). ^{13}C NMR (126 MHz, CHLOROFORM-d) δ 172.0, 154.7, 79.6, 45.5, 44.4, 42.7, 41.0, 28.2, 27.5, 24.0, 22.5, 22.4

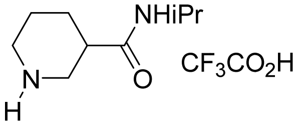

N-isopropylpiperidine-3-carboxamide trifluoroacetic acid salt: To a solution of tert-butyl 3-(isopropylcarbamoyl)piperidine-1-carboxylate (890 mg) in dichloromethane (5 ml) was added triisopropylsilane (250 µl) and trifluoroacetic acid (3 ml) at rt. After stirring for 24h at rt, the volatiles were removed *in vacuo* providing a viscous syrup used without further purification. ^{1}H NMR (500 MHz, METHANOL-d4) δ 3.96 (spt, J = 6.58 Hz, 1H), 3.15 - 3.29 (m, 3H), 3.02 - 3.14 (m, 1H), 2.68 (tt, J = 4.22, 8.27 Hz, 1H), 1.86 - 2.09 (m, 2H), 1.65 - 1.86 (m, 2H), 1.14 (d, J = 6.60 Hz, 3H), 1.15 (d, J = 6.60 Hz, 3H).

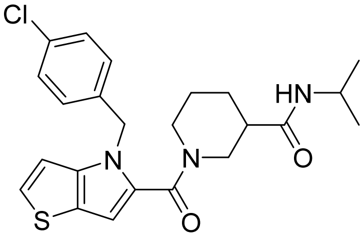

1-(4-(4-chlorobenzyl)-4H-thieno[3,2-b]pyrrole-5-carbonyl)-N-isopropylpiperidine-3-carboxamide (**2**):

General procedure for amide formation using HATU:

To a solution of 4-(4-chlorobenzyl)-4H-thieno[3,2-b]pyrrole-5-carboxylic acid (100 mg, 0.34 mmol, 1.0 equiv.) in anhydrous DMF (0.7 ml) was added *N,N*-diisopropylethylamine (0.24 ml, 1.37 mmol, 4.0 equiv.) followed by HATU (143 mg, 0.37 mmol, 1.1 equiv.) in one portion at rt. After stirring at rt for ca. 1 min., the solution of the activated ester was added to a vial containing N-isopropylpiperidine-3-carboxamide trifluoroacetic acid salt (146 mg, 0.51 mmol, 1.5 equiv.). After stirring at rt for 24h, the reaction was diluted with water, ethyl acetate and sat. aq. Na_2CO_3. The mixture was extracted with EtOAc (3 x 10 ml), and the organic layers were combined, washed with brine and dried with anhydrous $MgSO_4$. The volatiles were

removed in vacuo, and the resulting residue was absorbed onto silica gel and purified via silica gel flash chromatography (gradient 0-100% hexanes/ethyl acetate) to provide 124.5 mg of a pale-yellow foam (82% yield). ^{1}H NMR (500 MHz, METHANOL-d4) δ 7.31 (d, J = 5.35 Hz, 1H), 7.29 (d, J = 8.33 Hz, 2H), 7.08 (d, J = 8.33 Hz, 2H), 7.03 (d, J = 5.34 Hz, 1H), 6.70 (s, 1H), 5.35 - 5.55 (m, 2H), 4.33 (br. s., 1H), 4.19 (br. s., 1H), 3.93 (td, J = 6.64, 13.13 Hz, 1H), 3.01 (br. s., 2H), 2.23 (br. s., 1H), 1.88 (d, J = 12.42 Hz, 1H), 1.59 - 1.82 (m, 2H), 1.19 (br. s., 1H), 1.13 (d, J = 6.60 Hz, 7H). ^{13}C NMR (126 MHz, METHANOL-d4) δ 174.6, 165.0, 144.8, 138.6, 134.5, 130.7, 129.9, 129.9, 128.4, 123.7, 111.7, 106.1, 50.3, 44.5, 42.5, 29.2, 26.1, 22.7, 22.7 (missing one aliphatic ^{13}C signal). ^{13}C NMR (126 MHz, CHLOROFORM-d) δ 171.5, 162.8, 142.8, 136.6, 133.0, 129.1, 128.5, 128.4, 126.9, 122.0, 110.1, 104.7, 49.3, 46.3 (broad), 43.1, 41.1, 27.7, 24.5, 22.5, 22.4. MW: 443.99, Rt=2.73, M+1=444.0

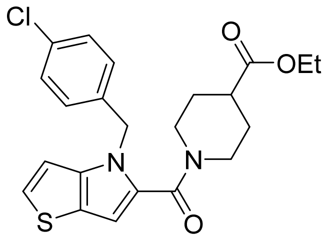

ethyl 1-(4-(4-chlorobenzyl)-4H-thieno[3,2-b]pyrrole-5-carbonyl)piperidine-4-carboxylate: ^{1}H NMR (500 MHz, CHLOROFORM-d) δ 7.21 - 7.25 (m, J = 8.49 Hz, 2H), 7.19 (d, J = 5.34 Hz, 1H), 7.04 - 7.11 (m, J = 8.33 Hz, 2H), 6.86 (d, J = 5.19 Hz, 1H), 6.57 (s, 1H), 5.43 (s, 2H), 4.29 (d, J = 11.95 Hz, 2H), 4.16 (q, J = 7.07 Hz, 2H), 3.03 (t, J = 11.40 Hz, 2H), 2.50 (tt, J = 3.91, 10.71 Hz, 1H), 1.84 (d, J = 11.16 Hz, 2H), 1.41 - 1.55 (m, 2H), 1.27 (t, J = 7.15 Hz, 3H), MW: 430.95, Rt=2.94, M+1=430.8

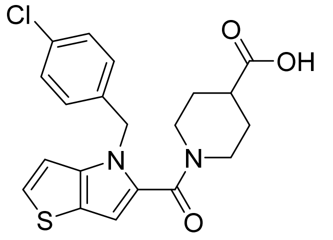

1-(4-(4-chlorobenzyl)-4H-thieno[3,2-b]pyrrole-5-carbonyl)piperidine-4-carboxylic acid: ^{1}H NMR (500 MHz, DMSO-d6) δ 12.29 (br. s., 1H), 7.40 (d, J = 5.34 Hz, 1H), 7.30 - 7.37 (m, J = 8.17 Hz, 2H), 7.21 (d, J = 5.19 Hz, 1H), 7.09 - 7.18 (m, J = 8.17 Hz, 2H), 6.70 (s, 1H), 5.44 (s, 2H), 4.10 (br. s., 2H), 3.02 (br. s., 2H), 1.74 (br. s., 2H), 1.29 (br. s., 2H). MW: 402.89, Rt=2.49 , M+1=402.9

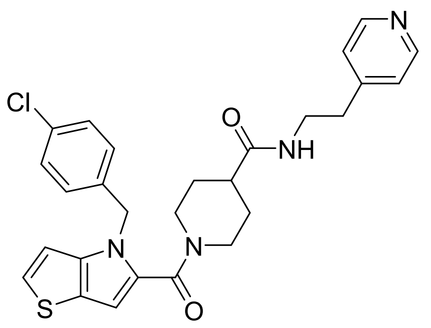

1-(4-(4-chlorobenzyl)-4H-thieno[3,2-b]pyrrole-5-carbonyl)-N-(2-(pyridin-4-yl)ethyl)piperidine-4-carboxamide (**9**): ^{1}H NMR (500 MHz, DMSO-d6) δ 8.45 (d, J = 4.87 Hz, 2H), 7.88 (t, J = 5.42 Hz, 1H), 7.39 (d, J = 5.19 Hz, 1H), 7.33 - 7.38 (m, J = 8.33 Hz, 2H), 7.18 - 7.24 (m, 3H), 7.13 - 7.18 (m, J = 8.17 Hz, 2H), 6.69 (s, 1H), 5.44 (s, 2H), 4.20 (br. s., 2H), 3.31 (s, 7H), 2.90 (br. s., 2H), 2.73 (t, J = 6.92 Hz, 2H), 2.32 (t, J = 11.00 Hz, 1H), 1.59 (d, J = 12.42 Hz, 2H), 1.35 (d, J = 12.26 Hz, 2H).

^{13}C NMR (126 MHz, CHLOROFORM-d) δ 173.8, 162.8, 150.0, 147.8, 142.8, 136.6, 133.3, 129.4, 128.7, 128.6, 126.8, 124.1, 122.1, 110.3, 104.4, 49.5, 43.1, 39.6, 35.0, 28.8 (missing 1 sp^3 ^{13}C signal).

Synthesis of indole analogs:

Scheme 2. Synthesis of Analogs 3 and 8

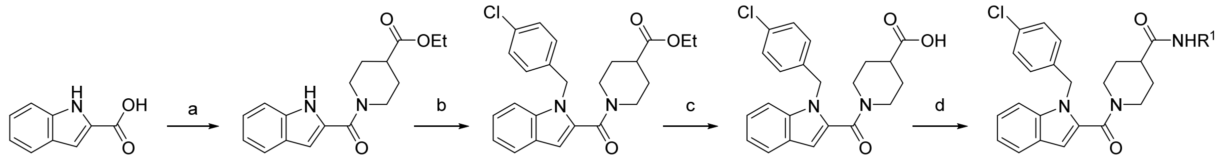

3, R^1 = iPr
8, R^1 = CH_2CN_2(4-py)

Reagents and conditions: (a) ethyl piperidine-4-carboxylate, HATU, DIEA, DMF, rt (b) Cs_2CO_3, 4-chlorobenzyl bromide, DMF, rt; (c) LiOH monohydrate, THF/MeOH/H_2O, rt (d) $NH_2CH_2CH_2$(4-py), HATU, DIEA, DMF, rt.

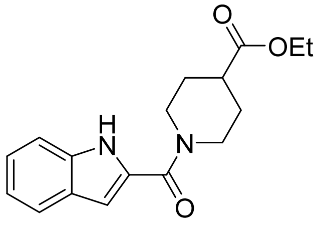

ethyl 1-(1H-indole-2-carbonyl)piperidine-4-carboxylate: To a solution of 1H-indole-2-carboxylic acid (3.02 g, 18.74 mmol, 1.0 equiv.) in anhydrous DMF (31 ml) was added ethyl piperidine-4-carboxylate (3.18 ml, 3.24 g, 20.61 mmol, 1.1 equiv.) and N,N-diisopropylethylamine (4.90 ml, 3.63 g, 28.11 mmol, 1.5 equiv.). After stirring at rt for 5 m, HATU (7.48 g, 19.67 mmol, 1.05 equiv.) was added in one portion at rt. After stirring at rt for 24h, the reaction was concentrated in vacuo via a rotary evaporator (bath temperature < 40° C). Water was added to the remaining residue, and the mixture was extracted with EtOAc (3 x 30 ml). The organic layers were combined and washed with sat. aq. Na_2CO_3, brine, and dried with anhydrous $MgSO_4$. The volatiles were removed in vacuo, and the resulting residue was re-crystallized in hot EtOAc and filtered to provide 4.079 g of a light tan solid (72% yield). ^1H NMR (500 MHz, CHLOROFORM-d) δ 9.02 (br. s., 1H), 7.65 (d, J = 7.86 Hz, 1H), 7.42 (d, J = 8.17 Hz, 1H), 7.29 (t, J = 7.62 Hz, 1H), 7.14 (t, J = 7.47 Hz, 1H), 6.73 - 6.81 (m, 1H), 4.56 (d, J = 13.99 Hz, 2H), 4.18 (q, J = 7.23 Hz, 2H), 3.28 (br. s., 2H), 2.56 - 2.72 (m, 1H), 1.98 - 2.11 (m, 2H), 1.75 - 1.91 (m, 2H), 1.28 (t, J = 7.07 Hz, 3H). ^{13}C NMR (126 MHz, CHLOROFORM-d) δ 174.1, 162.6, 135.8, 129.3, 127.3, 124.1, 121.7, 120.4, 111.8, 104.9, 60.7, 44.4 (broad), 41.0, 28.2, 14.2. R_t =2.48 min. ESI-MS m/z: $[M + H]^+$ 300.6.

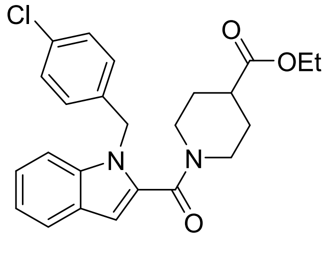

ethyl 1-(1-(4-chlorobenzyl)-1H-indole-2-carbonyl)piperidine-4-carboxylate: To a suspension of ethyl 1-(1H-indole-2-carbonyl)piperidine-4-carboxylate (507 mg, 1.68 mmol, 1.0 equiv.) and cesium carbonate (733 mg, 2.52 mmol, 1.5 equiv.) in anhydrous DMF (3.4 ml) was added 4-chlorobenzyl bromide (518 mg, 2.52 mmol, 1.5 equiv.) in one portion at rt. After stirring at rt for 18h, the reaction was diluted with water and extracted with EtOAc (2x20ml). The organic layers were combined, washed with brine, dried with anhydrous $MgSO_4$, and concentrated *in*

vacuo. Purification by silica gel flash chromatography (EtOAc/Hex) provided 734.2 mg of a colorless foam in 103% yield (EtOAc observed in ^{1}H NMR). ^{1}H NMR (500 MHz, CHLOROFORM-d) δ 7.65 (d, J = 7.86 Hz, 1H), 7.35 (d, J = 8.33 Hz, 1H), 7.26 - 7.30 (m, 1H), 7.19 - 7.23 (m, J = 8.49 Hz, 2H), 7.13 - 7.19 (m, 1H), 7.00 - 7.06 (m, J = 8.33 Hz, 2H), 6.63 (s, 1H), 5.48 (s, 2H), 4.60-3.90 (br. s, 4H), 4.16 (q, J = 7.18 Hz, 3H), 3.00 (br. s., 2H), 2.49 (tt, J = 4.03, 10.59 Hz, 1H), 2.05-1.15 (br. s, 4H), 1.26 (t, J = 7.15 Hz, 4H). ^{13}C NMR (126 MHz, CHLOROFORM-d) δ 173.9, 163.0, 137.5, 136.7, 133.3, 131.3, 128.7, 128.4, 126.5, 123.6, 121.7, 120.6, 110.0, 104.3, 60.7, 46.9, 40.9, 28.0, 14.2 (missing 1 sp^3 ^{13}C signal). R_t =3.08 min. ESI-MS m/z: $[M + H]^+$ 424.7.

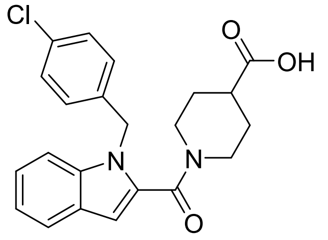

1-(1-(4-chlorobenzyl)-1H-indole-2-carbonyl)piperidine-4-carboxylic acid: To a mixture of ethyl 1-(1-(4-chlorobenzyl)-1H-indole-2-carbonyl)piperidine-4-carboxylate (ca. 734.2 mg, 1.68 mmol, 1.0 equiv.) in THF (1.7 ml), MeOH (1.7 ml) and water (1.7 ml) was added lithium hydroxide monohydrate (96 mg, 2.28 mmol, 2.0 equiv.) in in one portion at rt. After 18h of rapid magnetic stirring at rt, the starting material was consumed, and the organic solvents were removed *in vacuo*. The remaining aqueous solution was diluted with water (ca. 20 ml) and under vigorous magnetic stirring the pH was adjusted to pH=1-2 with the slow dropwise addition of conc. aq. HCl. A white precipitate formed during acidification and was collected via vacuum filtration, washed with water and dried under vacuum to provide 648.2 mg in 97% yield over 2 steps. The precipitate was used in the next step without further purification. ^{1}H NMR (500 MHz, CHLOROFORM-d) δ 7.65 (d, J = 7.86 Hz, 1H), 7.37 (d, J = 8.33 Hz, 1H), 7.26 - 7.32 (m, 1H), 7.19 - 7.23 (m, 2H), 7.13 - 7.19 (m, 1H), 6.99 - 7.07 (m, J = 8.49 Hz, 2H), 6.63 (s, 1H), 5.48 (s, 2H), 4.0-4.5 (m, 2H), 3.02 (br. s., 2H), 2.55 (tt, J = 4.01, 10.61 Hz, 1H), 1.0 – 2.0 (m, 4H). ^{13}C NMR (126 MHz, CHLOROFORM-d) δ 179.4, 163.1, 137.6, 136.6, 133.3, 130.9, 128.7, 128.5, 126.4, 123.7, 121.7, 120.6, 109.9, 104.5, 46.8, 41.3, 40.5, 27.7. R_t =2.65 min. ESI-MS m/z: $[M + H]^+$ 396.6, 398.6.

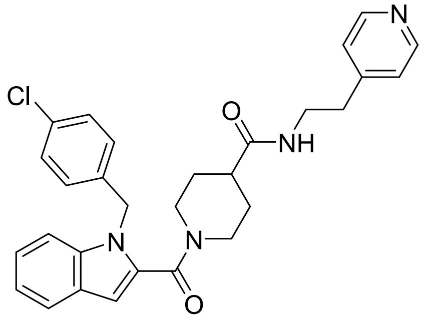

1-(1-(4-chlorobenzyl)-1H-indole-2-carbonyl)-N-(2-(pyridin-4-yl)ethyl)piperidine-4-carboxamide (**8**): To a solution of 1-(1-(4-chlorobenzyl)-1H-indole-2-carbonyl)piperidine-4-carboxylic acid (511.6 mg, 1.29 mmol, 1.0 equiv.) in anhydrous DMF (4.3 ml) was added *N,N*-diisopropylethylamine (337 µl, 1.93 mmol, 1.5 equiv.) followed by HATU (514.7 mg, 1.35 mmol, 1.05 equiv.) in one portion at rt. After stirring at rt for ca. 1 m, 4-(2-aminoethyl)pyridine (171 µl, 1.42 mmol, 1.1 equiv.) was added in one portion. After stirring at rt for 24h, the reaction was diluted with water, ethyl acetate and sat. aq. Na_2CO_3. The mixture was extracted with EtOAc (3 x 10 ml), and the organic layers were combined, washed with brine and dried with anhydrous $MgSO_4$. The volatiles were removed in vacuo, and the resulting residue was absorbed onto silica gel and purified via silica gel flash chromatography (gradient 100% hexanes/(ethyl acetate 20% MeOH, 1% Et_3N) to provide 631 mg of a white solid (97% yield). ^{1}H NMR (500 MHz, DMSO-d6) δ 8.45 (d, J = 5.19 Hz, 2H), 7.88 (t, J = 5.50 Hz, 1H), 7.63 (d, J =

8.02 Hz, 1H), 7.54 (d, J = 8.33 Hz, 1H), 7.34 (d, J = 8.33 Hz, 2H), 7.17 - 7.26 (m, 3H), 7.05 - 7.14 (m, 3H), 6.71 (s, 1H), 5.48 (s, 2H), 4.39 (br. s., 1H), 3.96 (br. s., 1H), 3.29 (s, 2H), 2.89 (br. s., 2H), 2.73 (t, J = 7.00 Hz, 2H), 2.24 - 2.39 (m, 1H), 1.61 (br. s., 2H), 1.36 (br. s., 3H). ^{13}C NMR (126 MHz, DMSO-d6) δ 173.5, 161.9, 149.3, 148.4, 137.2, 136.8, 131.9, 131.8, 128.8, 128.5, 126.2, 124.3, 123.1, 121.4, 120.2, 110.6, 103.4, 46.1, 45.8, 41.4, 38.8, 34.2, 28.3. A portion of this material was converted to the TFA salt for biological testing. R_t =2.35 min. ESI-MS m/z: $[M + H]^+$ 500.8, $[M + Na]^+$ 522.8, 524.6.

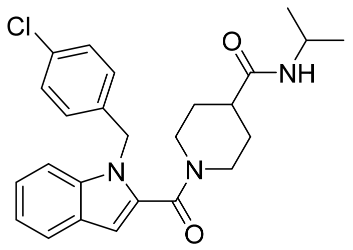

1-(1-(4-chlorobenzyl)-1H-indole-2-carbonyl)-N-isopropylpiperidine-4-carboxamide (**3**) was synthesized in a similar manner to that of 1-(1-(4-chlorobenzyl)-1H-indole-2-carbonyl)-N-(2-(pyridin-4-yl)ethyl)piperidine-4-carboxamide except isopropyl amine (1.25 equiv.) was used. Silica gel flash chromatography (0-20% methanol/chloroform) followed by C18 flash chromatography (0-100% acetonitrile (0.05% TFA)/water (0.05% TFA)). ^{1}H NMR (500 MHz, CHLOROFORM-d) δ 7.65 (d, J = 8.02 Hz, 1H), 7.29 - 7.36 (m, 1H), 7.27 (m, 1H), 7.19 - 7.24 (m, J = 8.49 Hz, 2H), 7.12 - 7.19 (m, 1H), 7.00 - 7.07 (m, J = 8.33 Hz, 2H), 6.65 (s, 1H), 5.47 (s, 2H), 5.19 (d, J = 8.02 Hz, 1H), 4.50 (br. s., 1H), 4.26 (br. s., 1H), 4.08 (qd, J = 6.58, 13.26 Hz, 1H), 2.87 (br. s., 2H), 2.23 (tt, J = 3.83, 11.18 Hz, 1H), 1.78 (br. s., 2H), 1.57 (br. s., 2H), 1.15 (d, J = 6.60 Hz, 6H). ^{1}H NMR (500 MHz, CHLOROFORM-d) δ 7.64 (d, J = 7.86 Hz, 1H), 7.30 - 7.36 (m, 1H), 7.27 (m, 1H), 7.18 - 7.24 (m, J = 8.33 Hz, 2H), 7.15 (t, J = 7.47 Hz, 1H), 6.99 - 7.07 (m, J = 8.33 Hz, 2H), 6.64 (s, 1H), 5.47 (s, 2H), 5.19 (d, J = 7.39 Hz, 1H), 4.37 (br. s., 1H), 4.08 (sxtd, J = 6.56, 13.56 Hz, 1H), 2.87 (br. s., 2H), 2.16 - 2.29 (m, 1H), 1.78 (br. s., 2H), 1.58 (br. s., 2H), 1.15 (d, J = 6.45 Hz, 6H). ^{13}C NMR (126 MHz, CHLOROFORM-d) δ .172.7, 163.0, 137.5, 136.6, 133.2, 131.3, 128.7, 128.3, 126.5, 123.6, 121.7, 120.6, 110.1, 104.3, 77.3, 76.7, 47.0, 43.1, 41.3, 28.7, 22.8 (missing 1 sp^3 ^{13}C signal).

Synthesis of pyrrole analogs:

Scheme 3. Synthesis of Analogs 4, 7, 10-12

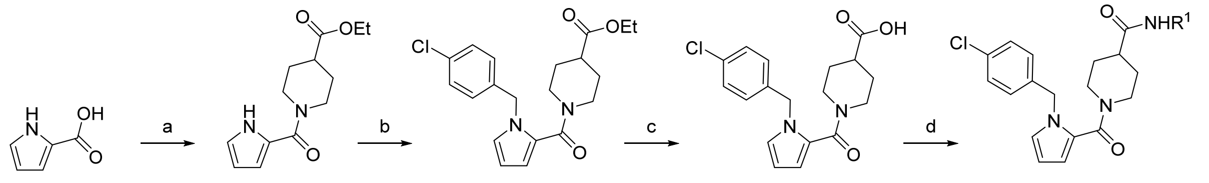

4, R^1 = iPr
7, R^1 = CH_2CH_2(4-py)
10, R^1 = CH_2(2-py) TFA salt
11, R^1 = CH_2(3-py) TFA salt
12, R^1 = CH_2(4-py) TFA salt

Reagents and conditions: (a) ethyl piperidine-4-carboxylate, HATU, DIEA, DMF, rt (b) Cs_2CO_3, 4-chlorobenzyl bromide, DMF, rt; (c) LiOH monohydrate, THF/MeOH/H_2O, rt (d) H_2NR^1, HATU, DIEA, DMF, rt.

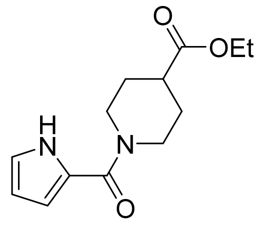

ethyl 1-(1H-pyrrole-2-carbonyl)piperidine-4-carboxylate: To a solution of pyrrole-2-carboxylic acid (0.96 g, 8.64 mmol, 1.0 equiv.), ethyl piperidine-4-carboxylate (1.46 ml, 9.50 mmol, 1.1 equiv.), and *N,N*-diisopropylethylamine (3.76 ml, 21.6 mmol, 2.5 equiv.) in 8.6 ml of THF was added 2,4,6-Tripropyl-1,3,5,2,4,6-trioxatriphosphorinane-2,4,6-trioxide solution (T3P) (7.2 ml of a 50wt% solution in EtOAc, 12.09 mmol, 1.4 equiv.) over 5m at rt. After stirring at rt for 18 h, the reaction was diluted with water and extracted with EtOAc (3x20ml). The organic layers were combined, washed with sat. aq. $NaHCO_3$, brine, dried with anhydrous $MgSO_4$, and concentrated *in vacuo*. Purification by flash chromatography provided 1.56 g of a white solid that contained ca. 5% impurity by ^1H NMR integration. The material was further purified via recrystallization (Hex/EtOAc) to provide 1.45 g of a white solid in 66% yield. ^1H NMR (500 MHz, DMSO-d6) δ 11.41 (br. s., 1H), 6.81 - 6.90 (m, 1H), 6.45 (br. s., 1H), 6.05 - 6.14 (m, 1H), 4.21 - 4.33 (m, 2H), 4.08 (q, J = 7.07 Hz, 2H), 3.09 (br. s., 2H), 2.65 (tt, J = 3.97, 10.89 Hz, 1H), 1.88 (dd, J = 3.38, 13.28 Hz, 2H), 1.41 - 1.60 (m, 2H), 1.19 (t, J = 7.07 Hz, 3H). ^{13}C NMR (126 MHz, DMSO-d6) δ 173.9, 161.5, 124.3, 121.0, 111.4, 108.3, 60.0, 43.6, 40.3, 28.1, 14.1. MW: 250.29, Rt=2.02, M+1=251.3

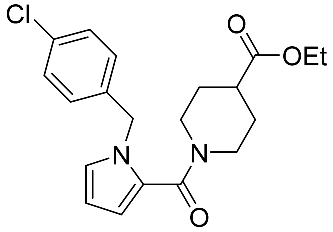

ethyl 1-(1-(4-chlorobenzyl)-1H-pyrrole-2-carbonyl)piperidine-4-carboxylate (**14**): To an ice water cooled solution of ethyl 1-(1H-pyrrole-2-carbonyl)piperidine-4-carboxylate (1.87g, 7.49 mmol, 1.0 equiv.) and 4-chlorobenzyl bromide (2.31g, 11.23 mmol, 1.5 equiv.) in anhydrous DMF was added small portions of NaH (ca. 95%, dry) until no further gas evolution was observed (ca. 375 mg NaH added). After stirring an addional hour at 0˚C, sat. aq. NH_4Cl was added dropwise to quench excess NaH. Once all the excess NaH had been quenched, the reaction was diluted with water and extracted with EtOAc (3x40ml). The organic layers were combined, washed with brine, dried with anhydrous $MgSO_4$, and concentrated *in vacuo*. Purification by flash chromatography provided 2.23 g of a clear colorless oil in 79% yield. ^1H NMR (500 MHz, CHLOROFORM-d) δ 7.20 - 7.31 (m, J = 8.33 Hz, 2H), 6.99 - 7.11 (m, J = 8.33 Hz, 2H), 6.76 - 6.85 (m, 1H), 6.32 (dd, J = 1.49, 3.69 Hz, 1H), 6.12 (t, J = 3.14 Hz, 1H), 5.28 (s, 2H), 4.18 - 4.35 (m, 2H), 4.15 (q, J = 7.18 Hz, 2H), 2.95 (t, J = 11.63 Hz, 2H), 2.46 (tt, J = 3.99, 10.71 Hz, 1H), 1.79 (d, J = 11.32 Hz, 2H), 1.32 - 1.55 (m, 2H), 1.26 (t, J = 7.15 Hz, 3H). ^{13}C NMR (126 MHz, CHLOROFORM-d) δ 174.0, 162.9, 137.0, 133.2, 128.6, 128.6, 125.4, 124.9, 112.8, 107.2, 60.6, 50.9, 44.4, 41.0, 28.0, 14.1; Rt=2.75, M+1=375.2

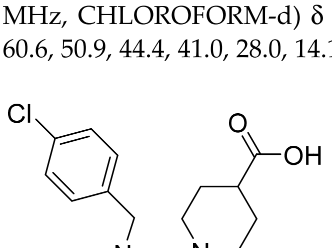

1-(1-(4-chlorobenzyl)-1H-pyrrole-2-carbonyl)piperidine-4-carboxylic acid (**13**): A similar procedure described for the synthesis of 4-(4-chlorobenzyl)-4H-thieno[3,2-b]pyrrole-5-carboxylic acid was followed to provide 6.00 g of **13** as a light tan crystalline solid in 91% yield. ^{1}H NMR (500 MHz, CHLOROFORM-d) δ 7.34 (d, J = 8.33 Hz, 2H), 7.01 - 7.15 (m, 3H), 6.30 (dd, J = 1.57, 3.62 Hz, 1H), 6.01 - 6.13 (m, 1H), 5.27 (s, 2H), 3.92 - 4.16 (m, 2H), 2.93 (br. s., 2H), 2.43 (tt, J = 4.01, 10.85 Hz, 1H), 1.69 (d, J = 10.53 Hz, 2H), 1.06 - 1.29 (m, 2H). MW: 346.81, Rt=2.25, M+1=347.0

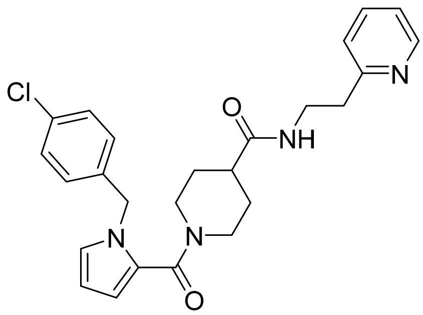

1-(1-(4-chlorobenzyl)-1H-pyrrole-2-carbonyl)-N-(2-(pyridin-2-yl)ethyl)piperidine-4-carboxamide (**5**) was synthesized using General procedure for amide formation using HATU and 2-(2-aminoethyl)pyridine to obtain 105 mg pale yellow solid in 65% yield. 1H NMR (500 MHz, CHLOROFORM-d) δ 8.46 (d, J = 4.40 Hz, 1H), 7.67 - 7.86 (m, 1H), 7.20 - 7.43 (m, 4H), 7.06 (d, J = 8.33 Hz, 2H), 7.01 (s, 1H), 6.37 (d, J = 2.20 Hz, 1H), 6.03 - 6.23 (m, 1H), 5.27 (s, 2H), 4.25 (br. s., 2H), 3.53 (t, J = 7.07 Hz, 2H), 2.96 (t, J = 7.00 Hz, 2H), 2.84 (br. s., 2H), 2.23 - 2.41 (m, 1H), 1.63 (br. s., 2H), 1.34 (br. s., 2H). 13C NMR (126 MHz, METHANOL-d4) δ 177.0, 165.3, 160.4, 150.0, 139.0, 138.8, 134.6, 130.1, 129.9, 127.3, 126.1, 125.3, 123.3, 114.5, 108.5, 51.8, 43.9, 40.4, 38.4, 29.9 (missing 1 sp^{3} ^{13}C signal).

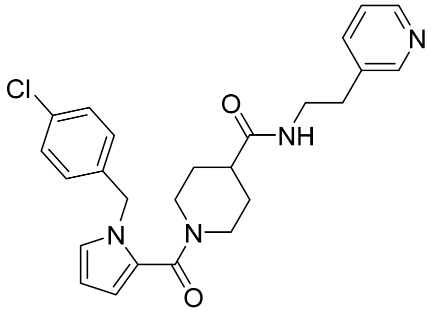

1-(1-(4-chlorobenzyl)-1H-pyrrole-2-carbonyl)-N-(2-(pyridin-3-yl)ethyl)piperidine-4-carboxamide (**6**) was synthesized using General procedure for amide formation using HATU and 3-(2-aminoethyl)pyridine to obtain 159.3 mg pale yellow solid in 98% yield. 1H NMR (500 MHz, CHLOROFORM-d) δ 8.38 (br. s., 2H), 7.72 (d, J = 7.70 Hz, 1H), 7.38 (dd, J = 5.11, 7.62 Hz, 1H), 7.21 - 7.34 (m, J = 8.49 Hz, 2H), 7.04 - 7.15 (m, J = 8.33 Hz, 2H), 7.01 (s, 1H), 6.26 - 6.49 (m, 1H), 6.04 - 6.23 (m, 1H), 5.27 (s, 2H), 4.25 (br. s., 2H), 3.43 (t, J = 6.92 Hz, 2H), 2.85 (t, J = 6.92 Hz, 4H), 2.32 (ddd, J = 3.77, 7.51, 11.36 Hz, 1H), 1.60 (br. s., 2H), 1.33 (br. s., 2H). 13C NMR (126 MHz, METHANOL-d4) δ 177.0, 165.3, 150.6, 148.2, 139.0, 138.9, 137.2, 134.6, 130.1, 129.9, 127.3, 126.0, 125.3, 114.5, 108.5, 51.8, 43.9, 41.3, 33.6, 30.0 (missing 1 sp^{3} ^{13}C signal).

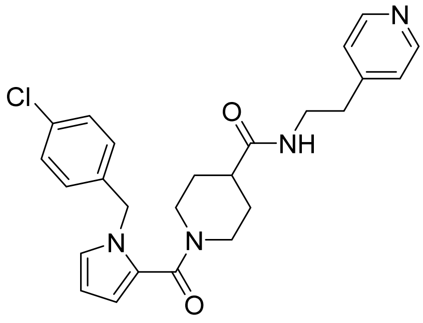

1-(1-(4-chlorobenzyl)-1H-pyrrole-2-carbonyl)-N-(2-(pyridin-4-yl)ethyl)piperidine-4-carboxamide (**7**) was synthesized using General procedure for amide formation using HATU and 4-(2-aminoethyl)pyridine to obtain 668.3 mg off white solid in 95% yield. 1H NMR (500 MHz, CHLOROFORM-d) δ 8.43 (d, J = 5.66 Hz, 2H), 7.22 - 7.43 (m, 4H), 7.07 (d, J = 8.33 Hz, 2H), 6.96 - 7.04 (m, 1H), 6.37 (dd, J = 1.57, 3.62 Hz, 1H), 6.05 - 6.25 (m, 1H), 5.27 (s, 2H), 4.25 (br. s., 2H), 3.46 (t, J = 7.00 Hz, 2H), 2.86 (t, J = 7.07 Hz, 4H), 2.32 (tt, J = 3.69, 11.40 Hz, 1H), 1.61 (br. s., 2H), 1.34 (br. s., 2H). 13C NMR (126 MHz, METHANOL-d4) δ 177.1, 165.3, 151.5, 150.1, 139.0, 134.6, 130.1, 129.9, 127.4, 126.2, 126.0, 114.5, 108.5, 51.8, 43.8, 40.6, 35.9, 29.9 (missing 1 sp^3 ^{13}C signal).

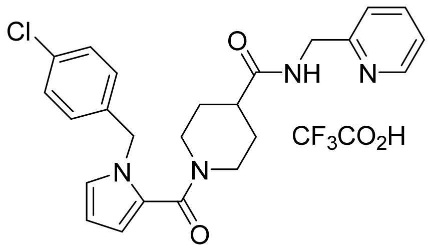

1-(1-(4-chlorobenzyl)-1H-pyrrole-2-carbonyl)-N-(pyridin-2-ylmethyl)piperidine-4-carboxamide trifluoroacetic acid salt (**10**) was synthesized using General procedure for amide formation using HATU and pyridin-2-ylmethanamine. The crude residue was purified via C18 flash chromatography (0-100% acetonitrile (0.05% TFA)/water (0.05% TFA)) to obtain 306.3 mg off a pink glass in 64% yield. Rt=2.24, M+=436.8. ^1H NMR (500 MHz, METHANOL-d4) δ 8.56 (d, J = 5.19 Hz, 1H), 8.03 (t, J = 7.78 Hz, 1H), 7.45 - 7.57 (m, 2H), 7.22 - 7.32 (m, J = 8.33 Hz, 2H), 7.03 - 7.10 (m, J = 8.33 Hz, 2H), 7.01 (s, 1H), 6.38 (d, J = 3.77 Hz, 1H), 6.14 (t, J = 3.14 Hz, 1H), 5.27 (s, 2H), 4.55 (s, 2H), 4.29 (br. s., 2H), 2.89 (br. s., 2H), 2.52 (tt, J = 3.71, 11.46 Hz, 1H), 1.63 - 1.87 (m, 2H), 1.27 - 1.51 (m, 2H).

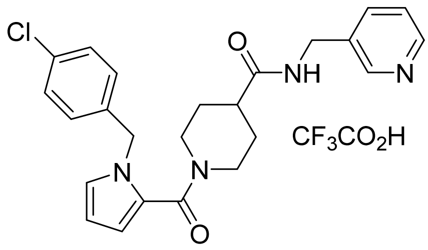

1-(1-(4-chlorobenzyl)-1H-pyrrole-2-carbonyl)-N-(pyridin-3-ylmethyl)piperidine-4-carboxamide trifluoroacetic acid salt (**11**) was synthesized using General procedure for amide formation using HATU and pyridin-3-ylmethanamine. The crude residue was purified via C18 flash chromatography (0-100% acetonitrile (0.05% TFA)/water (0.05% TFA)) to obtain 158.5 mg off a pink glass in 33% yield. Rt=2.21, M+=436.8. ^1H NMR (500 MHz, METHANOL-d4) δ 8.70 - 8.79 (m, 2H), 8.47 (d, J = 8.02 Hz, 1H), 7.95 - 8.04 (m, 1H), 7.23 - 7.31 (m, J = 8.17 Hz, 2H), 7.02 - 7.09 (m, J = 8.17 Hz, 2H), 7.01 (s, 1H), 6.32 - 6.41 (m, 1H), 6.14 (t, J = 2.99 Hz, 1H), 5.26 (s, 2H), 4.55 (s, 2H), 4.28 (br. s., 2H), 2.89 (br. s., 2H), 2.50 (tt, J = 3.60, 11.42 Hz, 1H), 1.63 - 1.84 (m, 2H), 1.23 - 1.47 (m, 2H).

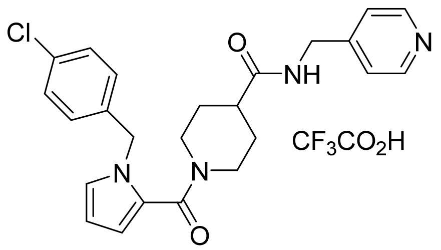

1-(1-(4-chlorobenzyl)-1H-pyrrole-2-carbonyl)-N-(pyridin-4-ylmethyl)piperidine-4-carboxamide trifluoroacetic acid salt (**12**) was synthesized using General procedure for amide formation using HATU and pyridin-4-ylmethanamine. The crude residue was purified via C18 flash chromatography (0-100% acetonitrile (0.05% TFA)/water (0.05% TFA)) to obtain 408 mg off a pale yellow solid in 85% yield. Rt=2.19, M+=436.8. ^{1}H NMR (500 MHz, METHANOL-d4) δ 8.70 (d, J = 5.82 Hz, 2H), 7.80 (d, J = 5.97 Hz, 2H), 7.23 - 7.34 (m, J = 8.33 Hz, 2H), 7.04 - 7.11 (m, J = 8.33 Hz, 2H), 7.02 (s, 1H), 6.33 - 6.46 (m, 1H), 6.14 (t, J = 3.07 Hz, 1H), 5.28 (s, 2H), 4.59 (s, 2H), 4.30 (br. s., 2H), 2.91 (br. s., 2H), 2.55 (tt, J = 3.67, 11.50 Hz, 1H), 1.70 - 1.88 (m, 2H), 1.33 - 1.41 (m, 2H).
